# Alimentary Tract Anatomy and Morphology in Early Adult Mediterranean Killifish *Aphanius fasciatus* (Valenciennes, 1821)

**DOI:** 10.3390/ani16040585

**Published:** 2026-02-12

**Authors:** Maria Cristina Guerrera, Lidia Pansera, Marialuisa Aragona, Kamel Mhalhel, Mauro Cavallaro, Maria Levanti, Rosaria Laurà, Giuseppe Montalbano, Francesco Abbate, Antonino Germanà

**Affiliations:** 1 Zebrafish Neuromorphology Lab, Department of Veterinary Sciences, University of Messina, Polo Universitario dell’ Annunziata, 98168 Messina, Italy; mariacristina.guerrera@unime.it (M.C.G.); lipansera@unime.it (L.P.); kamel.mhalhel@unime.it (K.M.); mauro.cavallaro@unime.it (M.C.); mblevanti@unime.it (M.L.); gmontalbano@unime.it (G.M.); abbatef@unime.it (F.A.); antonino.germana@unime.it (A.G.); 2 Department of Chemical, Biological, Pharmaceutical and Environmental Sciences, University of Messina, Viale Ferdinando Stagno D’Alcontres 31, 98166 Messina, Italy

**Keywords:** *A. fasciatus*, Mediterranean killifish, digestive tract, bioindicator species, experimental model, light microscopy, ecology, histological examination, tissue morphology

## Abstract

**Background/Aims:** The Mediterranean killifish, *Aphanius fasciatus* (Valenciennes, 1821), is a small euryhaline and eurytherm cyprinodont. While its ecology and role as a bioindicator are well known, its anatomy remains poorly understood. This study aimed to provide the first detailed description of the digestive tract of *A. fasciatus*, from the oropharyngeal cavity to the rectum. **Methods:** An anatomical and morphological approach supported by light microscopy was applied to examine oral, pharyngeal, and intestinal structures in adult specimens. **Results:** The jaws bear tricuspids incisiform teeth, whereas the pharynx has caniniform teeth. The tongue forms a muscular thickening of the oral floor and aids swallowing. The oropharyngeal tract and esophagus lead to a dilated anterior intestinal region resembling a primitive stomach, with mucosal folds and mucus-secreting epithelium, but lacking gastric glands. This chamber functions mainly for food storage, absorption, and preparation rather than true gastric digestion. The intestine extends to the rectum, showing sexual dimorphism: females have a relaxed anus, males a more toned and folded structure. **Conclusions:** These findings provide essential baseline information for comparison with other experimental models and reinforce the suitability of *A. fasciatus* as a reliable model for anatomical and functional studies.

## Simple Summary

The Mediterranean killifish, *Aphanius fasciatus*, is a small fish that is able to survive in variable salinity and temperature, making it an important bioindicator in coastal ecosystems. Despite its ecological relevance, little is known about its anatomy. This study provides the first detailed examination of its digestive tract, revealing a specialized anterior intestinal chamber that stores and processes food before digestion, and differences between males and females in the anal region. These findings offer valuable insight into the biology of this species and support its use as a reliable experimental model for studies in anatomy, physiology, and environmental monitoring.

## 1. Introduction

*Aphanius fasciatus* (Valenciennes, 1821), commonly known as the Mediterranean killifish, is a small teleost fish belonging to the order Cyprinodontiformes (Actinopterygii: Cyprinodontiformes). As a euryhaline and eurythermic species, it tolerates a wide range of physicochemical conditions, including temperature (5–39 °C) and salinity (0–180 g/L), allowing it to inhabit transitional environments such as estuaries and coastal lagoons [1,2,3,4]—shallow systems located between land and sea—along the Mediterranean region [5,6,7,8]. These habitats experience marked daily and seasonal fluctuations in physical and chemical properties, driven by tidal dynamics and solar heating [6,7,9]. *A. fasciatus* is typically associated with brackish or hypersaline water bodies, particularly salt marshes and lagoons, where environmental parameters show substantial variation [10,11,12]. The species completes its entire life cycle under these variable environmental conditions, reaching 2–9 cm in total length, reaching sexual maturity within the first year, with a lifespan of up to seven years [4,12,13,14,15]. Reproduction is characterized by strong male–male competition for access to females [16] and pronounced sexual dimorphism [16]. Females are generally larger than males but display less developed and less colorful caudal, anal, and dorsal fins [17]. In contrast, males exhibit bright body coloration and iridescent yellow pigmentation on the dorsal and anal fins, traits involved in intrasexual aggressive interactions [16,18]. The species exhibits rapid population dynamics due to its short generation time and high reproductive rate [8,14,16,18]. It is gregarious and largely sedentary, with limited dispersal capacity throughout the year. Seasonal movements are restricted, with populations likely shifting to deeper areas during winter and dispersing to nearby sites only during heavy rainfall events that flood coastal lagoons [12]. Consequently, gene flow among populations is limited, often resulting in marked genetic isolation even among geographically close groups [4,7,11,19]. Owing to these ecological characteristics, *A. fasciatus* has been widely employed as an indicator species for environmental quality in transitional waters, using molecular, cellular, and physiological biomarkers to assess contaminant effects [1,5,6,7,9,16,20,21,22]. In Italian transitional environments, only a limited number of fish species complete their entire life cycle within these habitats, among which the Mediterranean killifish *A. fasciatus* represents a key example [23]. Accordingly, Italian legislation recognizes this species as an indicator of complex ecosystems requiring specific conservation measures, in accordance with Council Directive 92/43/EEC. Despite the extensive ecological knowledge and its established role as a sentinel species, a comprehensive anatomical and morphological description of the gastrointestinal tract of *A. fasciatus* is still lacking. The present work addresses this gap, also revealing functional adaptations of the digestive system related to feeding habits and environmental variability. These fundings provide novel morphological data that support functional interpretations and comparisons with other teleost models used in biomedical and ecotoxicological research.

## 2. Materials and Methods

### 2.1. Sample Collection

**Figure 1:**
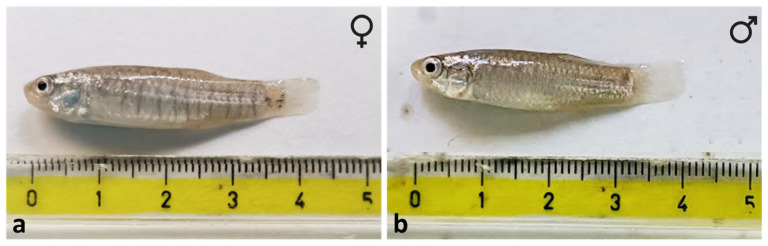
Representative specimens of *Aphanius fasciatus* collected at the study site, showing sexual dimorphism: (**a**) female, (**b**) male. Stereomicroscopic view.

A detailed macroscopic and microscopic morphological investigation was conducted on *Aphanius fasciatus* specimens in order to describe the species’ distinctive anatomical features and to highlight potential similarities with other experimental teleost models. Ten early adult *A. fasciatus* (five males and five females, with a body size ranging between 30 and 40 mm; see Figure 1) were collected in the Vendicari Nature Reserve (Siracusa, southeastern Sicily, Italy; 36.7907° N, 15.0896° E).

At the time of collection, ambient air temperature ranged approximately from 18 to 22 °C, relative humidity was moderate to high, and weather conditions were mild with no significant rainfall. The measured water physico-chemical parameters (Table 1) indicate a highly saline marine–brackish environment, well oxygenated and characterized by moderate turbidity, conditions consistent with habitats typically occupied by *Aphanius fasciatus*.

### 2.2. Light Microscopy

The specimens were fixed in Bouin solution for 12–18 h [24]; after removal of the excess fixative, they were maintained in ethanol 70° [25].

Macroscopic investigations were performed using a Leica stereomicroscope (M205C) equipped with a Leica IC80 HD digital camera (Leica, Milan, Italy) [25]. For histological purposes, the sample were dehydrated through a graded ethanol series (70–100°), cleared in xylene, and embedded in paraffin (Bio-Optica Milano S.p.A., Milan, Italy; code 08-7910) [26,27,28]. Paraffin blocks were sectioned into 7 μm serial slices using a Leica RM2135 microtome, mounted on gelatin-coated slides, and dried for 24 h [29,30,31,32]. The sections were subsequently deparaffinized, rehydrated, and stained with Masson’s trichrome using aniline blue (Cat.# 04-010802, Bio-Optica S.p.A., Milan, Italy) [33], and Alcian Blue pH 2,5-PAS (Bio-optica Milano S.p.A, Milan, Italy, CAT. # 04–163802) staining [27,34]. Finally, stained preparations were examined, and photomicrographs acquired with Leica Application Suite LAS v4.7 software using a Leica DMRB light microscope equipped with a Leica DFC7000 T camera (Leica Microsystems GmbH, Wetzlar, Germany) [35].

## 3. Results

**Figure 2:**
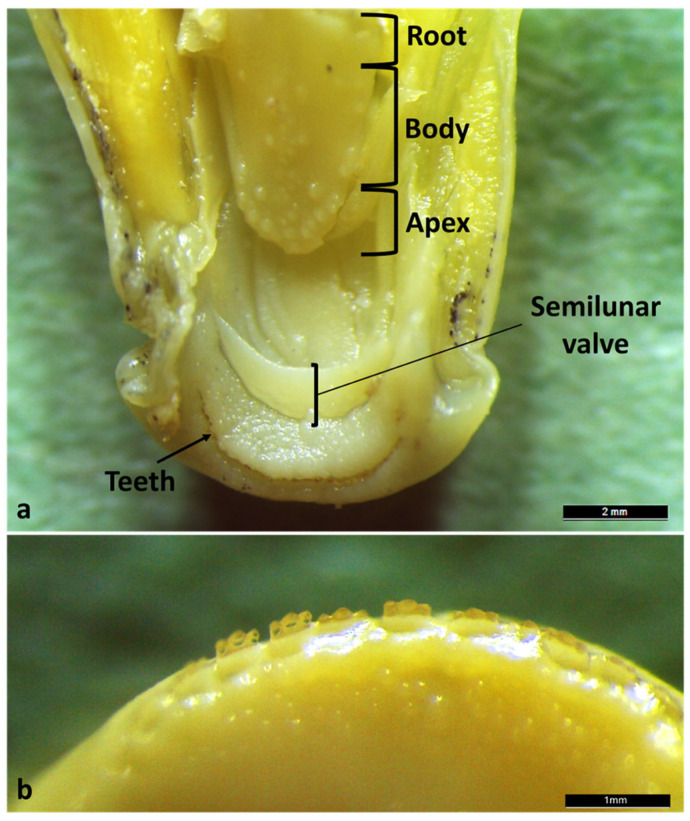
Mouth morphology of *A. fasciatus*. (**a**) Gross anatomy of jaws showing the tongue (apex, body, and root), the semilunar valve and teeth. (**b**) High magnification of tricuspid incisor-shaped teeth. Stereomicroscopic view.

The study examined the complete digestive tract of *Aphanius fasciatus* using longitudinal and transverse sections obtained from the entire specimen. To expose the tongue, the jaws were disarticulated. The tongue appears as a thickening of the floor of the mouth although it offers three regions for description: apex, body and root. Only the apex appears free (Figure 2a). On both jaws, the presence of a semilunar valve (Figure 2a) and a single row of small, tricuspid, incisive-like teeth were observed (Figure 2b).

**Figure 3:**
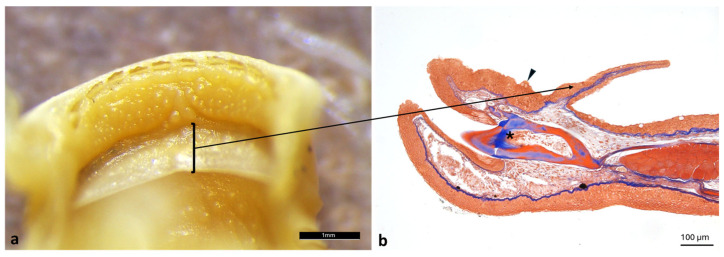
Semilunar valve morphology of *A. fasciatus*. (**a**) Stereomicroscopic view of the semilunar valve; the black square indicates the corresponding structure shown in the stained slide (**b**). (**b**) Masson’s Trichrome with Aniline Blue staining of the oral cavity showing the semilunar valve (arrow), taste buds (arrowhead), and the root of the tongue (asterisk).

Histological analysis of the semilunar valve revealed a thin connective core lined on both sides by a simple squamous epithelium interspersed with mucus-secreting goblet cells. The connective tissue is mainly composed of dense collagen fibers (Figure 3).

**Figure 4:**
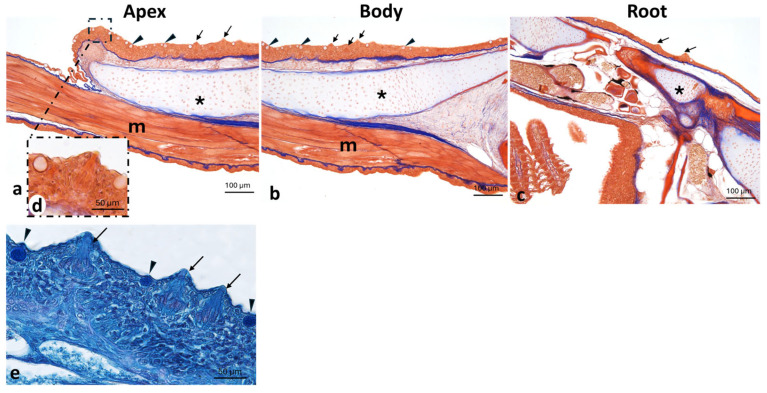
Mouth morphology of *A. fasciatus*. (**a**) Apex; (**b**) Body; (**c**) Root. Taste buds (arrows), goblet cells (arrowheads), cartilage (asterisk), muscle (m). (**d**) The inset shows a typical taste bud. (**e**) Taste buds (arrows) and goblet cells (arrowheads) revealed by Ab-PAS staining. Masson Trichrome with Aniline Blue staining.

Both the apex (Figure 4a) and the body (Figure 4b) are covered by mucosa lined with a non-keratinized stratified squamous epithelium containing abundant goblet cells and scattered taste buds, which progressively becomes thinner toward the root (Figure 4c). The submucosa, initially dense, progressively becomes looser and overlies a well-developed cushion of hyaline cartilage, which is continuous with the proper floor of the mouth, characterized by a prominent layer of striated muscle tissue (Figure 4).

**Figure 5:**
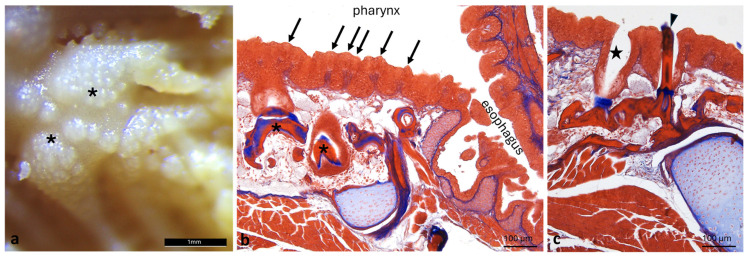
Pharynx morphology of *A. fasciatus*. (**a**) Stereomicroscopic view of the pharynx showing teeth (asterisks); (**b**) longitudinal section of pharynx showing taste buds (arrows) and teeth within the submucosae (asterisks); (**c**) erupted tooth (arrowhead), and empty alveolus following tooth exfoliation (star). (**b**,**c**) Masson’s trichrome with Aniline Blue staining.

Under the stereomicroscope, the surface of the pharynx does not appear smooth but features dense, irregularly distributed small elevations or protuberances (Figure 5a), which under the light microscope are identified as taste buds (Figure 5b). In the submucosa, pharyngeal teeth at different stages of eruption are present (Figure 5b,c). Some of them reach the surface (Figure 5c), and some dental alveoli are empty (Figure 5c).

**Figure 6:**
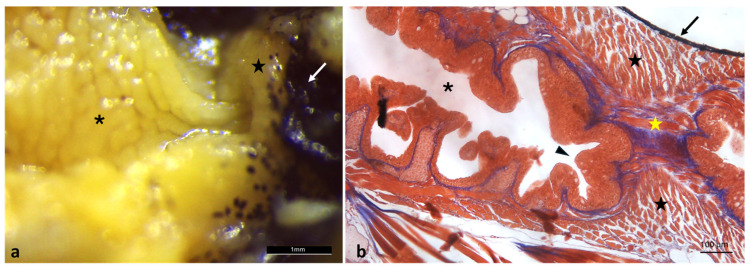
Pharyngeal region morphology of *A. fasciatus*. (**a**) Stereomicroscopic view of pharynx (asterisk) leading to the cardiac sphincter with pigmented ring (white arrow) and muscle (star); (**b**) Masson’s Trichrome with Aniline Blue staining of pharynx (asterisk) esophagus (arrowhead) and cardiac sphincter with pigmented ring (arrow), longitudinal muscle (yellow star), and circular muscle (stars).

The pharyngeal region continues posteriorly into the esophagus without a distinct structural boundary, leading to the cardiac sphincter. This area is marked by a distinctly pigmented ring (Figure 6a), while histological sections show circular muscle fibers indicative of sphincteric activity. The esophagus exhibits a mucosa raised in folds and lined by stratified squamous epithelium (Figure 6).

**Figure 7:**
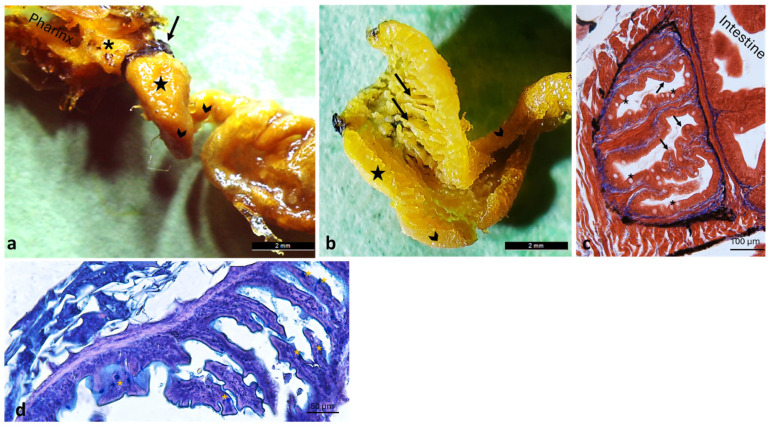
The intestinal tract of *A. fasciatus* originates at the pigmented cardiac sphincter (arrow) and continues into the pre-intestinal chamber. (**a**) Gross anatomy of the pre-intestinal chamber (star), the esophagus (asterisk), and the intestine (chevron arrows). (**b**) Gross anatomy of a cross-section of the pre-intestinal chamber (star) showing tall folds (arrows) leading into the intestine (black chevron arrows). (**c**) Masson’s Trichrome with Aniline Blue staining of the pre-intestinal chamber, showing tall folds (arrows) and a simple columnar epithelium interspersed with goblet cells (asterisks). (**d**) Pre-intestinal chamber, showing tall folds with a simple columnar epithelium and interspersed goblet cells (yellow asterisks) revealed by Ab-PAS staining.

Beyond the sphincter lies a balloon-shaped dilated portion, structurally adapted to facilitate the passage of food toward the intestine. This dilated region, referred to as the “pre-intestinal chamber”, exhibits a mucosa organized into tall folds and a simple columnar epithelium interspersed with mucus-secreting goblet cells (Figure 7).

**Figure 8:**
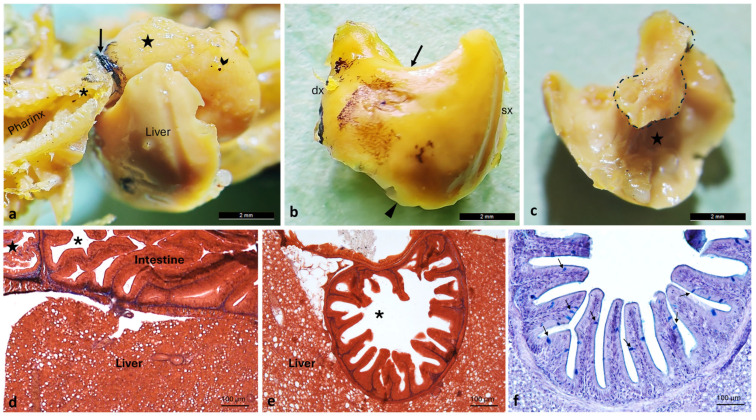
Liver morphology of *A. fasciatus*. (**a**) Stereomicroscopic view of the liver in situ, showing the esophagus (asterisk), the pigmented cardiac sphincter (arrow), the intestine (chevron arrow), and the pre-intestinal chamber (star). (**b**) Convex cranial surface of the liver, dorsal margin (arrow), ventral margin (arrowhead), right (dx) and left (sx) sides. (**c**) Concave visceral surface of the liver showing visceral impression (star); the hepatic lobe that surrounds the intestine in a latero-caudomedial direction is dashed. (**d**) Longitudinal section showing the pre-intestinal chamber (star), intestine (asterisk), and liver. (**e**) Transverse section of the intestine-encircling liver (asterisk). (**f**) Intestinal villi showing numerous goblet cells (arrows), as revealed by Ab-PAS staining. (**d**,**e**) Masson Trichrome with Aniline Blue.

The liver appears large, unilobed, and compact, its cranial margin is convex, whereas the dorsal margin is concave due to the impression made by the dilated intestinal portion (Figure 8). The liver partially surrounds the intestine through a small papillary process extending from the right margin, which curves caudomedially to partially encircle the proximal intestinal segment (Figure 8).

**Figure 9:**
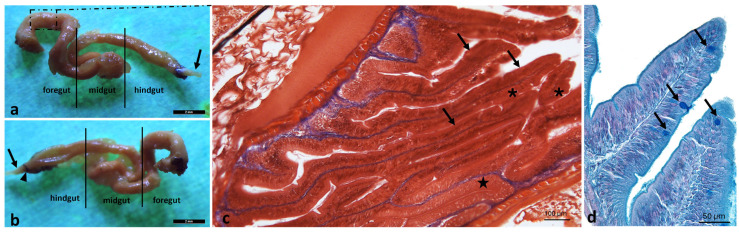
Intestine morphology of *A. fasciatus*. (**a**) Stereomicroscopic left lateral view showing the anterior intestine (foregut), middle intestine (midgut), posterior intestine (hindgut), and rectum (arrow). The inset indicates the intestinal region corresponding to the histological staining in (**c**). (**b**) Stereomicroscopic right lateral view showing the anterior intestine (foregut), middle intestine (midgut), posterior intestine (hindgut), and rectum (arrow); the rectal sphincter (arrowhead) is located at the junction between the posterior intestine and the rectum. (**c**) Masson’s Trichrome with Aniline Blue staining of the inset in (**a**) showing longitudinal folds of the foregut (arrows) lined by a simple columnar epithelium (star) with goblet cells (asterisks); connective tissue is stained blue. (**d**) Intestinal villi showing numerous goblet cells (arrows), as revealed by Ab-PAS staining.

The intestinal length is approximately four times the body length. It has a tubular shape and consists of the foregut, midgut, and hindgut; however, it lacks distinct regional segmentations, reflecting an omnivorous diet. The mucosa forms internal longitudinal folds that increase the absorptive surface area. The height and number of these folds gradually decrease in the caudal direction. A simple columnar epithelium interspersed with mucus-secreting goblet cells was observed (Figure 9).

**Figure 10:**
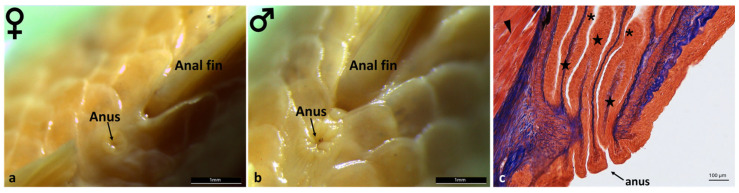
Morphology of the anal region of *A. fasciatus*. (**a**) In females, the anus appears relaxed, with mucosal folds that are faint or barely discernible (**b**) In males the anus exhibits a more tonic morphology, with tall and well-defined mucosal folds. (**c**) Masson’s Trichrome with Aniline Blue staining of the rectal tract showing the mucosa raised into folds (stars) lined by stratified squamous epithelium (asterisks) and supported by a dense connective tissue framework (blue staining). The folds extend into the anus, and a well-developed skeletal muscle layer is visible (arrowhead).

The morphological analysis of the anal region of *A. fasciatus* revealed marked sexual dimorphism. In females, the anus appears relaxed, with mucosal folds that are faint or barely discernible (Figure 10a). In males, by contrast, the anus exhibits a tonic morphology, with tall and well-defined mucosal folds (Figure 10b). Microscopic analysis of the rectal tract shows the mucosa raised into folds lined by stratified squamous epithelium, supported by a dense connective tissue framework. These folds extend into the anus. In addition, a well-developed skeletal muscle layer provides the sphincteric function (Figure 10c).

## 4. Discussion

Several studies have demonstrated the suitability of the Mediterranean killifish (*Aphanius fasciatus*) as a bioindicator species for environmental contamination in transitional waters, based on its molecular, cellular, and physiological responses to pollutants in estuaries and lagoons ecosystems [1,5,7,20]. Overall, these studies confirm the species’ sensitivity to environmental stressors and support its relevance for ecotoxicological monitoring. Within this framework, the present study provides the first comprehensive anatomical and morphological characterization of the gastrointestinal tract of *A. fasciatus*, from the oral cavity to the anal region, thereby establishing a structural framework for future functional, comparative, and toxicological investigations.

In the oral cavity, the presence of semilunar valves delimiting a depression identifiable as a gustatory fossa was observed. This structure, characterized by a high density of taste buds, likely functions as a pre-taste chamber. Histologically, the valves are composed of a simple squamous epithelium supported by connective tissue rich in dense collagen fibers, suggesting a structure capable of providing both stability and flexibility during feeding. Similar semilunar valves have been described in other teleosts, including *Danio rerio*, where upper and lower valves are prominent and lined by stratified epithelium containing numerous mucous and rodlet cells, indicating a conserved morphological feature involved in oral cavity partitioning and food manipulation [36]. Variations in valve morphology among teleost species are generally associated with different feeding strategies, such as suction feeding or substrate grazing [37,38]. In annual killifishes (Nothobranchius spp.), the oral region exhibits a simplified organization consistent with rapid life-history traits and opportunistic feeding, with reduced specialization compared with euryhaline species such as *A. fasciatus* [39]. In contrast, basal teleosts, including *Heterotis niloticus,* retain more archaic oropharyngeal structures, linked to omnivorous–detritivorous diets [25]. Compared with these species, *A. fasciatus* exhibits a more generalized oral configuration, supporting dietary flexibility in variable brackish environments.

The presence of small tricuspid, incisor-like teeth on both jaws represents a further distinctive feature of the anterior oral region of *A. fasciatus*. These teeth are continuously replaced, reflecting the polyphyodont condition typical of many teleosts [40]. Their morphology suggests a functional role in grasping and fragmenting food prior to swallowing. Comparable tricuspid dentitions have been reported in herbivorous and omnivorous teleosts, including *Ctenopharyngodon idella*, in which specialized pharyngeal teeth and a keratinized chewing pad are involved in processing of plant-derived material [41,42]. In contrast, carnivorous teleosts typically possess large conical or caniniform teeth adapted for prey capture and retention [43]. In *A. fasciatus*, the tricuspid morphology increases the functional surface area of the dentition, enhancing efficiency in cutting or grinding food prior to swallowing and indicating functional convergence with other euryhaline teleosts, including *Poecilia* spp. [44,45]. Conversely, *D. rerio* lacks oral teeth and relies exclusively on pharyngeal dentition located on the fifth ceratobranchials for suction-based feeding and crushing [40,46,47,48]. *H. niloticus* exhibits small conical (villiform) teeth arranged around a rigid, bone-supported “bony tongue,” forming a triturating surface adapted to omnivorous–detritivorous diets [25,49,50]. The tongue of *A. fasciatus* appears as a thickening of the floor of the mouth and is organized into three regions: the apex (partially free), the body, and the root. This tripartite organization parallels that described in *Oncorhynchus mykiss* and *Salmo salar*, where the tongue is associated with fungiform-like papillae, taste buds, and cartilaginous elements involved in food manipulation [35,51]. Similar epithelial features have been described in other teleosts, although the density and distribution of mucous cells and taste buds vary according to dietary habits [52,53,54]. Similarly, grass carp exhibits abundant taste buds on palatal and lingual folds supported by connective and muscular cores, reflecting adaptation to herbivorous feeding [39]. Compared with *Nothobranchius furzeri*, *A. fasciatus* shows a more differentiated lingual organization, likely related to its opportunistic feeding strategy in highly variable brackish environments [55,56]. In contrast, *H. niloticus* has a thick, cartilaginous “bony tongue” associated with tubular organs rich in mucous cells and taste buds, supporting extensive intraoral processing [25]. In adult *D. rerio*, the tongue is poorly regionalized, with a thinner epithelium and fewer mucus cells, reflecting suction-based feeding [57,58]. In this species, taste buds are widely distributed throughout the oral cavity, reducing the need for lingual specialization [57,59,60]. In *A. fasciatus*, the presence of scattered taste buds and goblet cells suggests an active role of the tongue in both sensory evaluation and lubrication of food. The hyaline cartilage pad underlying the lingual mucosa provides structural support that is absent in *D. rerio*, enhancing mobility during swallowing [58]. Accordingly, food transport in zebrafish relies mainly on hydrodynamic forces and branchial mechanisms rather than on active lingual movements [61,62,63,64,65]. The lingual mucosa of *A. fasciatus*, composed of non-keratinized stratified squamous epithelium with goblet cells and taste buds at an intermediate density, is consistent with an omnivorous feeding habit when compared with the higher densities reported in *Poecilia* spp. [44,45]. This organization, supported by a hyaline cartilage pad continuous with the striated musculature of the oral floor, parallels that described in grass carp and *Oncorhynchus* spp. [35,66,67], emphasizing functional versatility handling over specialized grazing in *Poecilia* spp. [13,15,44,45].

In line with the sensory and mechanical role of the oral structures, the pharyngeal region represents the functional continuation of food evaluation and processing. The pharyngeal tract of *A. fasciatus* is short and lined by stratified squamous epithelium with interspersed taste buds and well-developed pharyngeal teeth at different stages of eruption. The coexistence of sensory and masticatory components suggests a dual functional role, combining gustatory detection with mechanical food processing. Similar arrangements have been reported in other omnivorous teleosts, in which pharyngeal dentition contributes to crushing or grinding, while taste buds support food selection [37,68]. The presence of both oral and pharyngeal incisor-like teeth in *A. fasciatus* reflects adaptation to a mixed diet including algae, invertebrates, and larvae. Comparable patterns have been described in *O. niloticus* and *Ctenopharyngodon idella* [39,41,69], whereas strictly carnivorous species such as *Oncorhynchus* spp. possess larger conical pharyngeal teeth primarily adapted for prey retention rather than grinding [35]. In *D. rerio*, pharyngeal teeth are smaller and less robust, reflecting a diet dominated by microinvertebrates and detritus, while taste buds are widely distributed for chemical detection [58]. Nothobranchius spp. exhibit short pharyngeal tracts with simple dentition suited to rapid prey ingestion in ephemeral habitats [70]. Species of *Poecilia* spp. display moderate pharyngeal dentition adapted to both invertebrate- and plant-based diets [71], whereas *H. niloticus* shows robust pharyngeal teeth and a muscular tract associated with filter-feeding and food processing [25,49,50,72]. Overall, the integration of oral teeth, pharyngeal dentition, and taste buds enables *A. fasciatus* to exploit a wide range of trophic resources, linking its pharyngeal morphology to omnivorous feeding strategies across teleosts, from microprey specialists to dietary generalists and herbivores [73,74]. Once food processing within the oropharyngeal tract is completed, the digestive system transitions into the esophagus, a key segment responsible for controlled transport toward the intestinal tract.

Macroscopically, the cardiac region of the esophagus in *A. fasciatus* appears as a distinctly pigmented ring. Histological analysis identified this structure as composed of circular muscle fibers consistent with sphincteric function, regulating food transit from the oropharyngeal tract toward the digestive tract and preventing regurgitation. Well-developed esophageal sphincters are commonly reported in teleost fishes [75,76,77]. Distal to this region, the esophagus expands into a dilated balloon-shaped segment that likely serves as a temporary storage chamber prior to intestinal transit. Comparable esophageal dilations have been described in omnivorous species such as *O. niloticus* and *Ctenopharyngodon idella*, where they accommodate variable food volumes and facilitate peristalsis [39,75,76,78,79]. In contrast, *D. rerio* exhibits limited cardiac region dilation [80,81], while *Nothobranchius* spp. possess short esophagi with simple sphincters [82,83]. In *Poecilia* spp., sphincters are modest and dilation is reduced [84,85,86], whereas *H. niloticus* shows prominent sphincters and a markedly dilated proximal esophagus supporting bulk storage associated with filter-feeding [25,49,50,87]. The pronounced pigmentation of the cardiac ring in *A. fasciatus* may contribute to protection against mechanical stress or ultraviolet exposure, as suggested in other teleosts [25,75,88,89]. Together, the presence of both a functional sphincter and esophageal dilation represents an adaptive feature typical of omnivorous fishes, with structural variation reflecting different ecological strategies [76,90,91,92,93]. The anterior region of the digestive tract of *A. fasciatus* is characterized by a dilated chamber with tall mucosal folds lined by a simple columnar epithelium rich in mucin-secreting goblet cells, substantially increasing the absorptive surface area and facilitating pre-intestinal processing of ingested material [94,95,96,97,98]. The absence of true gastric glands indicates that this region functions as a predigestive tract, promoting mixing and partial absorption rather than acid or peptic digestion [99,100,101]. Agastric conditions represent a convergent evolutionary strategy among several teleost lineages [102,103]. In this context, the structure observed in *A. fasciatus*, here referred to as a “pre-intestinal chamber,” appears to optimize nutrient processing prior to enzymatic digestion. Comparable anterior intestinal enlargements, commonly described as “intestinal bulbs,” are reported in stomachless species such as *D. rerio* and other cypriniforms, where they represent major sites of enzyme activity and absorption [99,104,105,106]. Similar functional adaptations occur in *Nothobranchius* spp. [70,82,107,108] and *Poecilia* spp. [109,110,111], whereas *H. niloticus,* despite possessing a true stomach, exhibits proximal digestive segments showing functional similarities with predigestive chambers [25,87,112,113].

The functional efficiency of this predigestive region is closely supported by the associated hepatic system, which plays a key role in digestion and metabolic regulation.

Within the coelomic cavity, *A. fasciatus* present a large, compact, and unilobed liver. The organ displays a dorsal concavity produced by the impression of the proximal intestine, which is partially enveloped by a small hepatic process extending caudomedially from the right margin. This anatomical configuration highlights the central role of the liver in coordinating digestion and metabolism downstream of the predigestive tract [68,114,115]. Similar arrangements occur in Nothobranchius spp., in which the liver is closely associated with the short intestine, supporting rapid metabolic processing in ephemeral habitats [82,107,116]. In omnivorous *Poecilia* spp., the liver supports the metabolism of both animal and plant-derived nutrients [117,118,119]. *H. niloticus* features a large, lobed liver that supports bulk food storage and metabolic processing [25,87,112]. In agastric *D. rerio*, the liver is closely associated with the anterior intestinal bulb, contributing to bile secretion and metabolic regulation in the absence of a stomach [80,104,120]. Thus, *A. fasciatus* relies on an integrated hepatic system to support digestion through bile production and metabolic regulation, paralleling teleosts with agastric or partially agastric digestive tracts. Following hepatic secretion and metabolic processing, the digestive tract continues into the intestine, which represents the main site of digestion and nutrient absorption in this agastric species.

The intestine in *A. fasciatus* is tubular and lacks distinct regional segmentation, consistent with an omnivorous feeding regime. The mucosa forms longitudinal folds lined by a simple columnar epithelium with interspersed mucin-secreting goblet cells [13,80,102,114,115,121,122]. In the absence of a stomach, enteric digestion, supported by early pancreatic and biliary secretions, replaces gastric processing [106,114,123]. The relative length of the intestine exceeds that typically observed in predatory species, supporting the digestion of a mixed diet that includes plant material, detritus, and invertebrates [92,124]. Folds enhance digestive efficiency without increasing organ volume, paralleling simplified digestive models such as those of *D. rerio* and *Poecilia reticulata*, which rely on decentralized digestive functions [118]. Poecilia spp. shows long, folded intestine compensating for absent stomachs, processing plant and animal matter [85,125]. In contrast, Nothobranchius spp. exhibit shorter intestinal tracts adapted for rapid ingestion of invertebrates [82,107,108,126]. *H. niloticus* exhibits a long, highly folded intestine integrating digestive secretions to support bulky omnivory [25,49,50,72]. Thus, *A. fasciatus* relies on extended intestinal absorption and digestive secretions distributed along the intestinal tract, consistent with omnivory in agastric teleosts.

Finally, the morphological analysis of the anal region in *A. fasciatus* revealed marked sexual dimorphism. Females exhibited a lax anus with barely evident or absent mucosal folds, reflecting reduced sphincter tonicity that likely facilitates egg passage during oviposition in externally fertilizing species [15,16,127,128]. In contrast, males showed a tonic anus with prominent mucosal folds, indicating enhanced sphincter musculature development that allows controlled milt release, enhancing reproductive efficiency [15,128,129]. Comparable sexual dimorphism is observed in other externally fertilizing teleosts. In *D. rerio*, males display distinctive anal fin pigmentation and genital pore morphology associated with spawning behavior [80,130,131,132,133]. In *P. reticulata*, males possess a gonopodium derived from the anal fin, enabling directed sperm transfer, whereas females retain a more flexible oviposition region [134,135,136]. Males of *Nothobranchius* spp. exhibit hypertrophied anal musculature adapted for rapid milt expulsion, while females maintain compliant anal regions suitable for brief spawning events [82]. In *H. niloticus*, sexual dimorphism of the anal region is less pronounced but still reflects differential sphincter development linked to gamete control and egg passage [25,136]. Overall, this dimorphism in *A. fasciatus* underscores the specialization of the anal region for sex-specific reproductive roles, correlating mucosal architecture and sphincter tonicity with fertilization efficiency across teleosts.

## 5. Conclusions

This study provides the first comprehensive anatomical and histological description of the digestive tract of *Aphanius fasciatus*, offering a detailed overview of its structural organization and adaptive features.

The integration of macroscopic and microscopic observations reveals a digestive system well suited to a broad range of environmental conditions, consistent with the species’ remarkable euryhaline nature. The structural traits observed suggest functional specializations that enhance digestive efficiency and physiological plasticity under fluctuating salinity and nutrient availability. From an ecological perspective, these findings shed light on how *A. fasciatus* maintains digestive performance in habitats exposed to anthropogenic stressors, thus reinforcing its recognized resilience in transitional and brackish environments. The morphological data contribute to comparative frameworks within teleost digestive biology, highlighting both conserved features shared with cyprinodontiforms and distinctive traits that reflect the adaptive evolution of this taxon. Moreover, the detailed dataset provided here offers a solid foundation for future functional, molecular, and ecotoxicological investigations, enabling meaningful comparisons with other teleost model species and helping to distinguish conserved digestive traits from lineage- and habitat-specific adaptations. From a translational viewpoint, the integration of ecological, anatomical, and ecotoxicological information in *A. fasciatus* supports its role as a bridge species between environmental risk assessment and experimental pathophysiology, particularly for investigating pollutant-induced alterations of digestive structure and function.

Given its tolerance to environmental stress and its ease of maintenance under laboratory conditions, this species represents a suitable model for evaluating the effects of contaminants on digestive physiology, as well as for comparative anatomical studies among euryhaline teleosts. Collectively, the evidence presented confirms the scientific relevance of *A. fasciatus* as both a bioindicator species and as an experimental model organism in biology research. Moreover, this study focused on early adult subjects, so the anatomical patterns reported should be interpreted within this context. Considering that data from other developmental stages are currently missing in bibliography, these results lay the groundwork for future comparable studies on the developmental changes in the gastrointestinal tract in *A. fasciatus*.

## Figures and Tables

**Table 1:** In situ physico-chemical parameters of the water measured at the sampling site.

Parameter	Symbol	Value	Unit	Notes
pH electrode potential	mVₚₕ	−62.5	mV	Raw electrode signal
pH	pH	8.13	–	Alkaline water
Oxidation–reduction potential	ORP	93.8	mV	Weakly oxidizing conditions
Dissolved oxygen	DO	103.0	% sat.	Supersaturation
Electrical conductivity	EC	70.90	mS·cm^−1^	Measured value
Electrical conductivity (25 °C)	EC_25_	70.83	mS·cm^−1^	Temperature-compensated
Resistivity	ρ	0.0000	MΩ·cm	Inverse of conductivity
Salinity	Sal	35.45	ppt	Parts per thousand
Practical salinity	PSU	48.63	PSU	Practical Salinity Units
Density (sigma-t)	σₜ	33.7	–	Relative seawater density
Turbidity	χ	24.95	NTU	Moderate turbidity

## Data Availability

All data presented in this study are available from the corresponding author upon responsible request.

## Abbreviations

*A. fasciatus*	*Aphanius fasciatus*
*H. niloticus*	*Heterotis niloticus*
*D. rerio*	*Danio rerio*
*O. niloticus*	*Oreochromis niloticus*
*P. reticulata*	*Poecilia reticulata*
LAS	Leica Application Suite
dx	right
sx	left
m	muscle
